# Cognitive Involvement in Balance, Gait and Dual-Tasking in Aging: A Focused Review From a Neuroscience of Aging Perspective

**DOI:** 10.3389/fneur.2018.00913

**Published:** 2018-10-29

**Authors:** Karen Z. H. Li, Louis Bherer, Anat Mirelman, Inbal Maidan, Jeffrey M. Hausdorff

**Affiliations:** ^1^Department of Psychology, Concordia University, Montreal, QC, Canada; ^2^Centre for Research in Human Development, Concordia University, Montreal, QC, Canada; ^3^PERFORM Centre, Concordia University, Montreal, QC, Canada; ^4^Department of Medicine, Université de Montréal, Montreal, QC, Canada; ^5^Centre de Recherche de l'Institut Universitaire de Gériatrie de Montréal, Montreal, QC, Canada; ^6^Research Center, Montreal Heart Institute, Montreal, QC, Canada; ^7^Center for the Study of Movement, Cognition and Mobility, Neurological Institute, Tel Aviv Sourasky Medical Center, Tel Aviv, Israel; ^8^Department of Neurology, Sackler Faculty of Medicine and Sagol School of Neuroscience, Tel Aviv University, Tel Aviv, Israel; ^9^Department of Physical Therapy, Sackler Faculty of Medicine and Sagol School of Neuroscience, Tel Aviv University, Tel Aviv, Israel; ^10^Rush Alzheimer's Disease Center and Department of Orthopaedic Surgery, Rush University Medical Center, Chicago, IL, United States

**Keywords:** gait, balance, aging, cognitive training, dual task, cognition, motor-cognitive interference

## Abstract

A substantial corpus of evidence suggests that the cognitive involvement in postural control and gait increases with aging. A large portion of such studies were based on dual-task experimental designs, which typically use the simultaneous performance of a motor task (e.g., static or dynamic balancing, walking) and a continuous cognitive task (e.g., mental arithmetic, tone detection). This focused review takes a cognitive neuroscience of aging perspective in interpreting cognitive motor dual-task findings. Specifically, we consider the importance of identifying the neural circuits that are engaged by the cognitive task in relation to those that are engaged during motor task performance. Following the principle of neural overlap, dual-task interference should be greatest when the cognitive and motor tasks engage the same neural circuits. Moreover, the literature on brain aging in general, and models of dedifferentiation and compensation, in particular, suggest that in cognitive motor dual-task performance, the cognitive task engages different neural substrates in young as compared to older adults. Also considered is the concept of multisensory aging, and the degree to which the age-related decline of other systems (e.g., vision, hearing) contribute to cognitive load. Finally, we discuss recent work on focused cognitive training, exercise and multimodal training of older adults and their effects on postural and gait outcomes. In keeping with the principle of neural overlap, the available cognitive training research suggests that targeting processes such as dividing attention and inhibition lead to improved balance and gait in older adults. However, more studies are needed that include functional neuroimaging during actual, upright performance of gait and balance tasks, in order to directly test the principle of neural overlap, and to better optimize the design of intervention studies to improve gait and posture.

## Introduction

Approximately 30% of individuals over age 65 experience one or more falls each year (1, 2), leading to significant health care costs worldwide (3). Accumulating behavioral, neuropsychological, and neuroimaging evidence shows that slow gait, postural instability, and fall risk are associated with cognitive capacity. More specifically, poor mobility in aging has been associated with exaggerated effects of cognitive-motor dual tasking, cognitive impairment, and degeneration of gray and white matter in anterior brain regions that subserve executive functions (EFs) and link to motor regions (4–8). Substantial progress has been made in understanding the cortical control of gait and balance, with several comprehensive reviews on this topic (9, 10).

Inasmuch as cognitive contributions to posture and gait are well-acknowledged in the movement sciences, there is less consideration of the basic literature on age differences in patterns of neural activity during cognitive performance, and the potential for cognitive and neural plasticity through training in old age to ameliorate the age-associated declines. These basic aging findings suggest that older adults commonly activate additional brain regions when performing cognitive tasks, compared to young adults, suggesting that cognitive involvement in motor behaviors may have different implications for older adults than for younger adults. Nonetheless, we suggest that a greater consideration of findings from the cognitive neuroscience of aging can enhance the interpretation of two major experimental paradigms: (1) the cognitive-motor dual-task paradigm, which aims to restrict or occupy the available cognitive capacity hypothesized to support motor functioning in old age and assess the impact on performance; (2) the cognitive remediation or training paradigm, which aims to enhance available cognitive capacity and/or increase neural efficiency, and thereby free up cognitive resources to support motor functioning.

**Figure 1:**
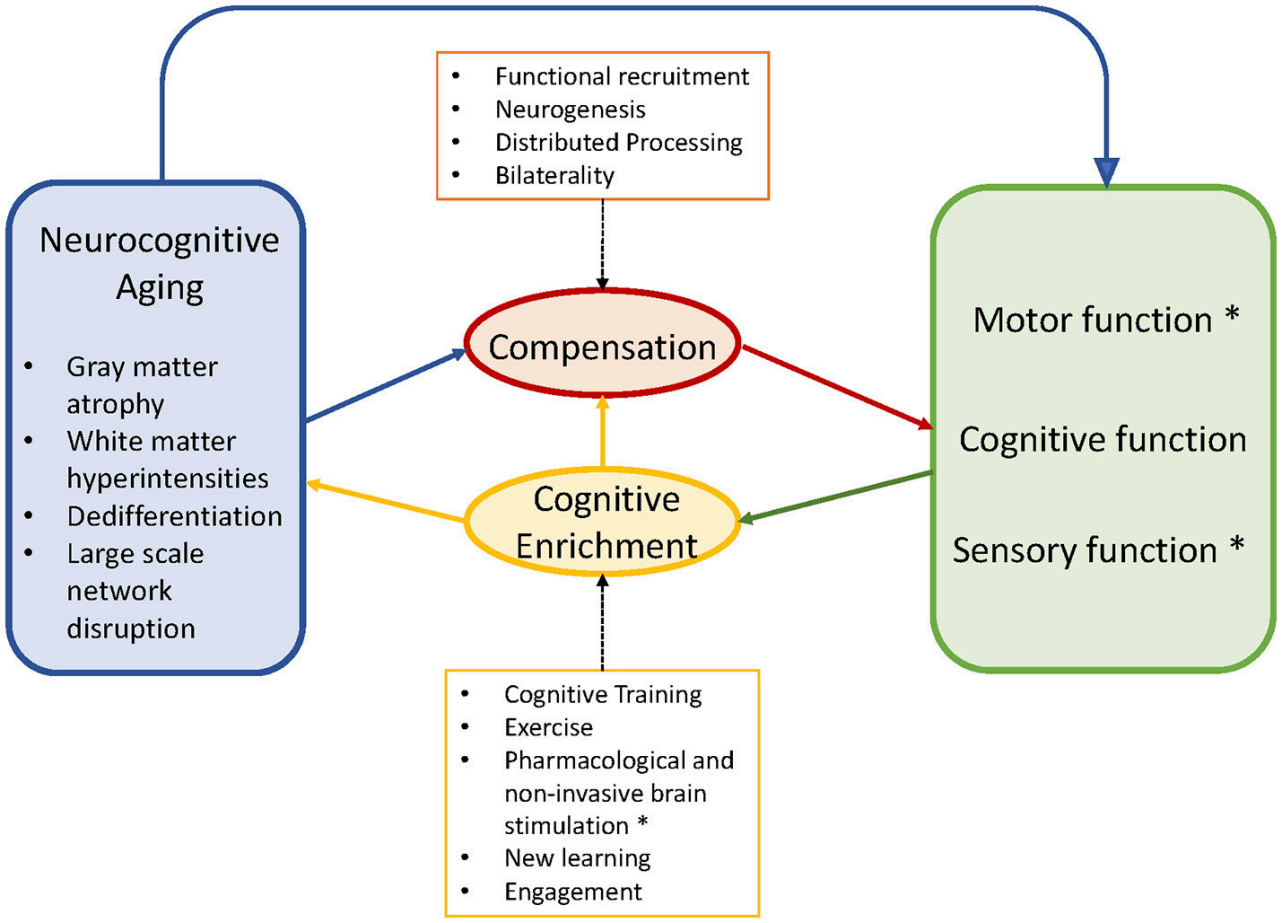
Conceptual diagram adapted from Park and Reuter-Lorenz (11). Asterisks indicate our modifications and extensions, particularly the inclusion of motor and sensory functioning. The schematic shows the negative aspects of neurocognitive aging (blue) that trigger compensation via functional recruitment in older adults (red). These maladaptive and adaptive factors jointly contribute to observed motor, cognitive, sensory functions (green). Cognitive enrichment (yellow) can ameliorate aspects of brain aging and facilitate compensatory efficiency if there is neural overlap between the improved and targeted outcomes. Motor, cognitive, and sensory functions benefit from, and compete for, common capacity (e.g., prefrontal cortex), particularly during complex behaviors such as cognitive-motor dual-tasking. Copyright permission not required.

Accordingly, in this review, we first describe current findings in neurocognitive aging, with an emphasis on empirical evidence of cognitive processes that have been related to postural control and gait. We then discuss major models that link neural aging with plasticity and compensatory patterns of neural activity, such as the Scaffolding Theory of Aging and Cognition [STAC: (11)] and Hemispheric Asymmetry Reduction in Older Adults [HAROLD: (12)]. We then summarize the research on cognitive plasticity in an effort to contextualize the recent application of cognitive training or dual-task training to improve gait and posture. We then review recent empirical work on cognitive-motor dual-tasking (CMDT), and on cognitive training and associated mobility gains. Finally, we consider the implications from the cognitive neuroscience of aging work as applied to the study of gait and posture. Figure 1 illustrates the proposed joint influences of neurocognitive aging and compensation on cognitive capacity, the implications for cognitive, perceptual and motor performance, and the potential for cognitive enrichment to improve these performances.

## Neurocognitive aging

Brain aging has been well-described in terms of both structural and functional dimensions (13), multiple chapters; (9). Briefly, longitudinal studies indicate that cortical gray matter volumes decrease linearly across adulthood (from the 20s to 80s) in frontal and temporal regions, whereas other regions such as primary visual, parietal, and entorhinal cortex remain relatively stable across adulthood (14). Regions such as the dorsolateral prefrontal cortex (DLPFC) and the orbitofrontal cortex appear to be most age-sensitive (15), and are associated with concomitant behavioral declines in cognitive functions such as working memory, episodic memory encoding, and divided attention (13). Other regions with accelerated decline in aging include the cerebellar hemispheres and the hippocampus (16). Diffusion tensor imaging (DTI) reveals normative changes in white matter volume and integrity with aging, such that anterior white matter tracts show greater age-related declines as compared to more posterior tracts [e.g., (17)].

In the adult lifespan, these white matter changes precede gray matter volumetric declines, thus compromising the efficiency of communication between regions (11). White matter hyperintensities (WMH), an index of lesion burden, explain more of the age-related variance in cognitive performance than total brain volumes (11). Recent work suggests a negative relationship between white matter integrity and functional activation, as if to compensate for the white matter decline, coined the “less wiring more firing” principle (18, 19).

## Compensation

Functional neuroimaging studies of cognitive aging, using primarily MRI or PET, commonly reveal age-related increases in frontal activation bilaterality during tests of memory, which are associated with better cognitive performance [e.g., (20, 21)]. Another notable pattern, observed in older compared to younger adults, is prefrontal up-regulation during memory encoding, coupled with reduced parahippocampal activation, suggesting a compensatory function for the extra neural recruitment in response to decreased activity in the task-relevant brain regions (22). Similarly, in auditory tasks such as speech perception in the presence of noise, older adults exhibit greater activity in frontal regions and less activity in auditory cortex (23), possibly reflecting the recruitment of intact cognitive functions such as verbal ability and semantic memory (24). Notably, the observed increase in neural recruitment among older adults may only be effective in mitigating the negative effects of neural aging to a point. In a study of aging and *n*-back working memory performance, older adults performed as well as younger adults (1-back) and showed greater bilateral prefrontal activity (BA 9) during that condition. However, with greater levels of memory load (2- and 3-back), older adults performed worse than younger adults and did not exhibit increased prefrontal activity (25), suggesting that a functional limit of compensatory recruitment had been reached.

Notably, not all extra activations are considered adaptive. Dedifferentiation, or loss of neural specificity, has been observed in the visual cortex where activations are more diffuse in older than younger adults [e.g., (26)]. These functional imaging observations are mirrored in behavioral observational studies of sensory and sensorimotor abilities, which appear to share increasing variance with many cognitive functions in older age (27, 28). Similarly, older adults exhibit ability dedifferentiation (29) within EF measures (inhibition, updating, switching) that are identified in young adulthood as relatively distinct factors (30).

Finally, studies of functional connectivity suggest that the dynamic coordination of large-scale networks is disrupted with aging, potentially leading to the observed cognitive decline [e.g., (31, 32)]. It appears that older adults recruit the fronto-parietal and salience networks less consistently than young adults (33), resulting in diminished frequency of switching between large-scale networks and reduced flexibility in performance (34). Age-related decline in white matter integrity and gray matter volume are correlated with activity in prefrontal nodes of the salience and fronto-parietal network, possibly a consequence of compensatory mechanisms (33).

Together, these negative attributes of brain aging (e.g., gray and white matter changes, dedifferentiation, and large scale network disruption) have been conceptualized as complementary to the observed compensatory patterns of brain activity (e.g., frontal recruitment, bilaterality) in models of cognitive aging such as the STAC model [(11); STAC-R: (35)], and HAROLD model (12). Both models propose that upregulation of additional brain regions occurs in response to age-related neurodegeneration, and that older adults who do not exhibit such upregulation tend to exhibit lower levels of cognitive performance than those who do. Notably, the potentially positive, compensatory patterns of neural recruitment take place in the same regions that show the greatest degeneration with aging. However, the research on cognitive enrichment (36, 37) offers encouragement in terms of potential for the improvement of EFs. In the STAC models, the capacity to engage in compensatory scaffolding is enhanced through cognitive training, social stimulation, and exercise (11, 35).

## Cognitive enrichment

While a detailed review of the topic of cognitive enrichment in aging research is beyond the scope of the present review [see (36, 37)], we highlight a few key issues that are of relevance for the involvement of cognitive aging in balance and gait. A central issue in this research domain is the extent to which older adults can improve through cognitive training, and if the trained skill(s) transfer to untrained skills. Process-based cognitive training studies, in which targeted cognitive mechanisms are trained via computer programs, commonly show that healthy older adults exhibit robust gains in the trained cognitive processes or skills (37, 38). However, older adults do not exhibit significant transfer beyond the trained tasks unless there is an overlap in underlying processes [e.g., (39)]. By contrast, greater transferability of trained skills is observed in studies that target EFs such as divided attention, working memory, and task switching (40–43).

The question of when to expect broader transfer of training can be addressed with the Principle of Neural Overlap (36, 42, 44). This principle proposes that the degree of common neural activation between trained and untrained cognitive tasks should correspond to the degree of training-related transfer observed. This is illustrated well in Dahlin's study of EF training and transfer (42). Young and older adults were trained for 5 weeks on a memory updating task engaging the striatum, as shown in pre-training fMRI scans. The researchers also assessed an array of transfer tasks that showed varying levels of functional and neural overlap with the trained updating task. In the young adults, behavioral evidence of transfer was greatest for another updating task (*n*-back) associated with striatial activation, and less evident for those outcomes engaging other neural structures. In contrast, older adults showed no training-related transfer to the *n*-back task, nor was there any striatal activation during this task.

Kramer's early studies of dual-task training (45) showed greater training-related transfer to other variants of dual-task performance when a variable priority procedure (emphasis on two cognitive tasks; Task A vs. B changed across blocks) was used, compared to a fixed priority procedure involving equal attention allocation between tasks [see also (46, 47)]. Importantly, although older adults can be trained to divide attention accurately between two tasks when instructed, the allocation of attention between tasks of differing priority (48) or difficulty level (49) in more ecological contexts may differ from that observed in the laboratory.

The extant evidence on cognitive and neural plasticity appears promising [see (36, 50) for reviews] and relevant to mobility. Lövden et al. (51) compared young and older adults after roughly 100 h of training using multiple cognitive tasks (working memory, episodic memory, processing speed), reporting significant white matter improvement (FA) in the older adults, particularly in the anterior portion of the anterior cingulate cortex (ACC). Behavioral changes were observed in working memory, perceptual speed and episodic memory, although the degree of improvement was differentially greater in younger adults only for perceptual speed and episodic memory. In an fMRI study (52), using Bherer's dual-task training protocol, young adults showed significant pre-to-post increases in bilateral DLPFC activity while performing the trained task, and the degree of activation change correlated with the degree of improvement in behavioral reaction time data for the dual-task condition. In follow-up work, older adults showed a training-related increase in left VLPFC and a decrease in DLPFC bilaterally, suggesting a shift to using articulatory rehearsal (i.e., subvocal repetition of task-relevant information) as a control strategy (53). Finally, functional neural changes appear to precede structural changes, occurring after as little as 9 h of multimodal cognitive training in older adults (54).

A second major strategy of cognitive enrichment is exercise training. As reviewed elsewhere (55, 56), training studies have abundantly demonstrated that moderate aerobic exercise such as walking, swimming, or cycling, improves attentional control and executive functioning in older adults, compared to non-aerobic protocols such as stretching or toning (57–59). Strength or resistance training can also benefit cognitive ability and brain health (55, 60), but evidence for its impact on multiple EFs is presently limited in comparison to the aerobic training findings. Neuroplastic changes after aerobic training echo the behavioral cognitive findings in showing increased efficiency in brain regions associated with executive control processes. For example, (61) compared older adults assigned to an aerobic exercise versus a stretch control condition on fMRI during flanker task selective attention performance. The aerobic group showed improved attentional control, and increased task-related activity in right middle frontal gyrus and superior parietal regions. The aerobic group also showed greater volumetric increases in anterior white matter, gray matter in left inferior frontal gyrus, anterior cingulate, and superior temporal gyrus (62). Using DTI, (63) found increases in the white matter integrity (fractional anisotropy: FA) of prefrontal and temporal regions in older adults and associated improvements in short-term memory after aerobic exercise (walking) but not toning. Aerobic training appears to trigger global neuroplastic effects by increasing the production of neurotrophic factors (e.g., BDNF, IGF-1, VEGF) that are able to cross the blood-brain barrier and support neurogenesis, vascularization, axonal repair, and synaptogenesis, particularly in frontal, prefrontal, and hippocampal regions [Erickson (64)]. Notably, levels of these neurotrophic factors were positively associated with exercise-induced increases in temporal lobe functional connectivity (65).

A recent meta-analysis examined combined, multi-modality training protocols and cognitive outcomes (66). Compared to exercise training alone, combined exercise and cognitive training appears superior in the majority of aging studies [e.g., (67)], whereas multi-modal training is not consistently superior to cognitive training alone [e.g., (68)].

Together, the extant findings on neurocognitive aging and cognitive enrichment reveal a number of observations that are relevant for our understanding of postural control and gait in aging. First, neuroimaging findings suggest that the health of white matter tracts and functional connectivity between brain regions may be more age-sensitive than volumetric or functional assessments of discrete brain regions. We note that while many earlier studies focused on the relationships between changes in the structure of specific brain regions and their impact on behavioral outcomes like CMDT, more recent work emphasizes the connections across networks; structural changes in the PFC, for example, may reverberate across a relatively large attention network that extends beyond the PFC. Second, age-related dedifferentiation, coupled with compensatory scaffolding, suggests that there may be qualitative differences in neural activation and behavioral strategies exhibited in young versus older adults. Third, cognitive training research suggests that targeting specific EFs, and including a flexibility or variable-priority component, yields the broadest transfer to untrained cognitive skills. However, an important qualification is that, following the principle of neural overlap, training efficacy apparently depends on the degree of neural similarity between trained and untrained skills. Similarly, the degree of interference observed during dual-tasking should depend on the degree of competition for common neural structures.

We turn next to recent findings in cognitive-motor dual-tasking and aging, focusing on the neural underpinnings of single-task gait and balance as well as CMDT. The fact that CMDT induces activation of cognitive and motor networks simultaneously, both networks that share common pathways as well as very specific pathways, enables the examination of their vulnerability during cognitive-motor interference and the compensatory mechanisms that are called into play with aging.

## Cognitive reduction: cognitive-motor dual-tasking (CMDT)

The ability to divide attention between cognitive and motor activity has been examined in efforts to quantify the amount of cognitive capacity recruited for motor functioning (8, 69). CMDT designs typically contrast a balance or walking task performed alone (single task), versus the same motor task performed with a concurrent cognitive task (e.g., talking, mental arithmetic). This comparison forms the basis for the calculation of dual-task costs (DTCs: [single - dual task]/single), which indicate the degree of performance decline or cognitive recruitment that is prompted by the secondary task. Cognitive recruitment to aid motor performance is presumed to reduce the already limited cognitive capacity that can be devoted to a concurrent cognitive task in old age (28, 48).

Briefly, the behavioral CMDT research on postural control shows that age-related DTCs are exacerbated by a variety of factors such as postural threat (70), reduced sensory inputs (71), platform perturbations (72), and concurrent visual imagery (73). Similarly, in studies of gait, age-related increases in DTCs are observed when walking over obstacles (74), and with increased complexity of walking (75). Simple cognitive loads may elicit dual-task motor facilitation relative to no-load conditions, while more complex cognitive loads elicit proportionately greater costs in postural stability (76) and in an array of spatiotemporal gait parameters (77). In light of the known frontal recruitment associated with age-related sensory decline, it is perhaps not surprising that additional competition for cognitive capacity is observed when auditory challenges are experimentally imposed on dual-task walking (78) and dual-task balance (79), or when older adults with hearing impairment undergo CMDT (80).

### Structural brain changes associated with gait, balance, and CMDT

Studies of brain structure and mobility provide convergent evidence for the cognition-mobility link in older adults. For example, gray matter volumes in the left DLPFC were correlated with usual gait speed in healthy older adults, whereas reduced volumes in putamen and superior posterior parietal lobule were associated with balance difficulty during semi-tandem stance (81). Interestingly, the association between prefrontal volumes and gait speed was mediated by cognitive processing speed (82). In Parkinson's disease (PD), which has been viewed to some degree as a model of “unsuccessful” aging, structural and functional MRI were used to compare PD fallers and non-fallers (83). The fallers, as compared to non-fallers, showed reduced volumes in the caudate head region of the basal ganglia, coupled with increased resting state connectivity in posterior parietal regions of the central executive network. In general, the DTCs that occur in aging are more exaggerated in the presence of neurodegenerative disease, like PD [see (84) for a recent review]. This pattern of reduced structural integrity in task-specific brain regions, coupled with increased neural recruitment in other areas, is consistent with the STAC model, in that prefrontal recruitment is considered a compensatory response to neural degeneration of conventionally relevant networks.

Age-related changes in white matter integrity have also been implicated in mobility status. Moscufo et al. (85) observed associations in healthy older adults between gait speed and WMH burden in the anterior corpus callosum (splenium), attributing the reduction of mobility to a disruption of interhemispheric transfer of visual and somatosensory information. Similarly, Srikanth et al. (86) reported that bilateral frontal periventricular white matter lesion volumes correlated with a composite gait score, and attributed this association to a disconnection from frontal motor cortical areas with subcortical regions (e.g., basal ganglia). Similar to Rosano et al. (82) gray matter results, Bolandzadeh et al. (87) found that the relationship between WMHs in frontal corpus callosum and gait speed was mediated by cognitive processing speed. Everyday levels of physical activity also appear to moderate the relationship between global WMH burden and mobility (88), similar to the cognitive findings (63). Ezzati et al. (89) reported significant associations between cortical gray matter volumes and usual gait speed, but not with total white matter or ventricular volumes. In contrast, in the LADIS study of patients presenting with mild memory complaints (90, 91), the severity of age-related white matter changes was associated with the severity of gait and functional decline. Using DTI methods (FA), Bruijn et al. (92) reported significant associations in older adults between parameters of gait quality during treadmill walking and the diffusivity of the left anterior thalamic radiation (connecting the thalamus to frontal regions).

### Functional imaging of gait and balance

There is a growing number of functional brain imaging studies during walking [for reviews: (93, 94)]. Imagined walking and dual-task walking during fMRI scans elicit activations in supplementary motor (SMA) and prefrontal regions in older adults, but show less activity in the same brain regions than what is observed during actual walking as measured using portable neuroimaging methods (95). Using fMRI and motor imagery during imagined walking, compared to watching a film, PD patients had greater activation in inferior frontal gyrus and precuneus than healthy controls [Maidan (96)]. These findings suggest that there is increased recruitment of frontal regions in PD even during imagined walking; perhaps this is an ineffective compensatory attempt. In a study of healthy older adults, resting state functional connectivity in sensorimotor, visual, vestibular, and left fronto-parietal regions was associated with off-line gait velocity under single-task and walking while talking CMDT conditions (reciting alternating letters of the alphabet), and supplementary motor and prefrontal regions were associated with only with CMDT (97).

**Figure 2:**
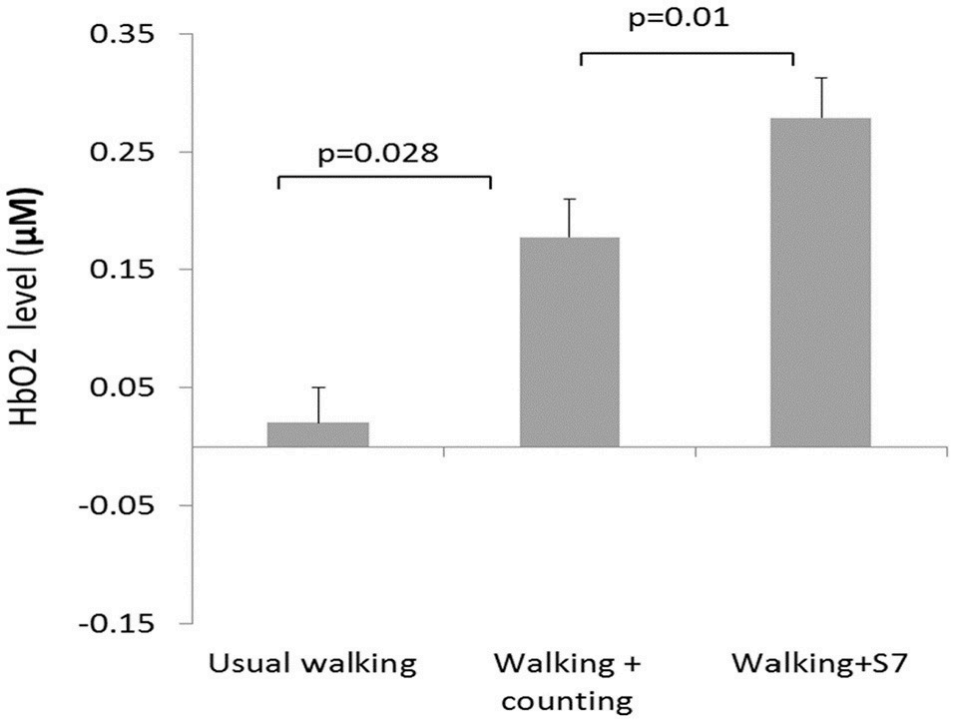
*fNIRS* results (102) showing significant increases in blood oxygenation (HbO_2_) as a function of walking complexity, i.e., the greater the cognitive load, the greater the increase in frontal activation during walking. Copyright permission not required.

**Figure 3:**
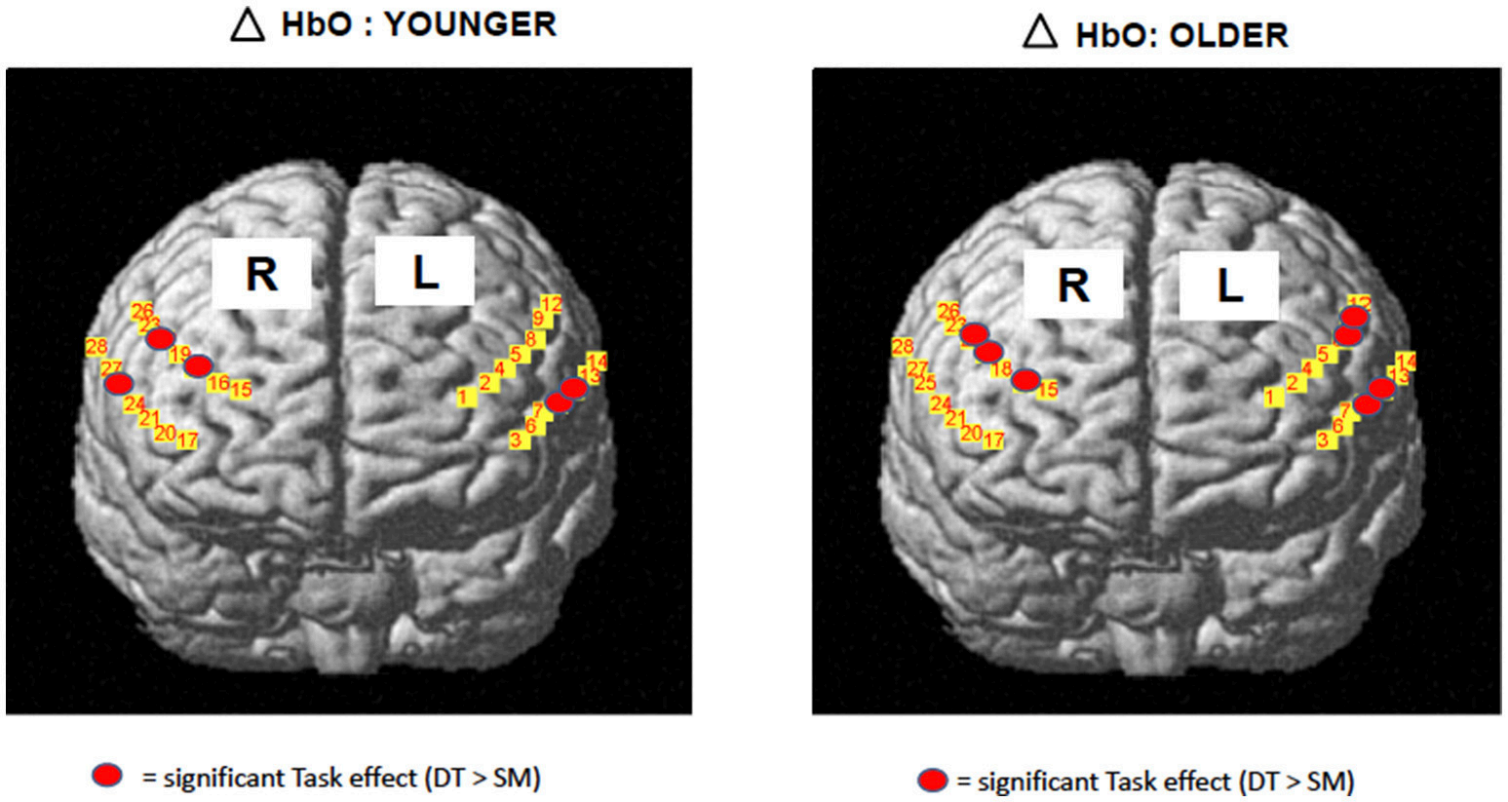
*fNIRS data* [adapted from (103)] 19 young, 14 older adults during treadmill walking alone or with *n*-back cognitive load. Both age groups showed greater bilateral HbO_2_ change from single motor to dual task conditions, OA had greater bilateral activations. Copyright permission not required.

The development of portable functional near-infrared spectroscopy [fNIRS: (98)] allows brain activity to be measured during active, upright (not just imagined) walking. Like fMRI, fNIRS directly measures the hemodynamic response (oxy- & deoxygenated hemoglobin: HbO_2_, HbR), and is more tolerant of motion artifacts (99). In early studies of fNIRS and dual-task walking, Holtzer et al. (100, 101) reported greater prefrontal activation overall (HbO_2_) in younger versus older adults, and increased HbO_2_ during dual-task walking while talking compared to walking alone in young adults. By contrast, older adults showed less of a dual-task change, and their HbO_2_ levels did not correlate with walking speed, suggesting that older adults show less efficient or effective frontal recruitment compared to young adults. In a recent study of young adults, a graded positive association between rostral frontal cortex HbO_2_ levels (Brodmann's area 10) and increasingly complex dual-task contexts (walking alone, with counting forward, and with serial 7s subtraction) was observed (see Figure 2) (102). Others recently reported that activity in bilateral prefrontal cortices increased with cognitive load (*n*-back auditory working memory) during walking (103). Further, younger adults showed more left lateralization whereas older adults showed more bilateral activity (see Figure 3), consistent with the HAROLD model (12). In contrast, (104) found decreased activation of the prefrontal cortex under CMDT conditions (walking + visual checking) relative to single-task walking, and this downregulation was associated with greater dual-task costs in walking. The authors surmise that older adults were recruiting other posterior regions during complex CMDT as an alternative to prefrontal cortex, although their fNIRS array did not permit empirical confirmation. Using a dynamic balance protocol with varying sensory inputs (somatosensory, visual, vestibular) and fNIRS, others have shown that both middle-aged and older adults increase their frontal-lateral blood oxygenation during dynamic balance tasks (105).

Another recent development in the gait literature is the assessment of walking using electroencephalography [EEG: (10, 106)]. Notably, spectral power analyses have revealed that the gamma band is sensitive to manipulations of attentional load during walking in older adults and in neurological patients. More gamma band activity (30–100 Hz), which is associated with attention, learning, and memory (107), was observed in neurological patients than in healthy older adults (106), providing convergent evidence for increased cognitive compensation. Functional neuroimaging studies of balance, reviewed elsewhere (10) and in this special issue, reveal a similar pattern. For example, in EEG studies using postural evoked potentials and platform translations, the N100 amplitude, associated with attentional orienting responses, commonly shows attenuation in response to perturbations and cognitive loads such as visuomotor tracking or visual memory [e.g., (108, 109)].

In sum, the extant findings on neuroimaging, aging, and mobility suggest that many of the age-normative brain changes associated with declining cognitive control are associated with diminished gait and postural control. The literature on online neuroimaging of gait implicates prefrontal, premotor, and SMA regions (93). Under more challenging conditions involving CMDT, younger adults appear to recruit left prefrontal regions whose activity is related to better performance, whereas older adults show more bilateral recruitment that is not as well-linked to performance.

## Cognitive enhancement

Interventions designed to improve CMDT performance have focused largely on practicing the targeted motor function alone, termed “specific single-task training,” or when combined with a cognitive load, termed “specific dual-task training” (110). This is contrasted with training on related but non-matching motor tasks, termed “general single- or dual-task training.” The latter is recommended to strengthen attentional flexibility and dual-task management [e.g., (47, 111–113)]. The present focus, however, is to review training studies that more directly evaluate the cognitive contributions to balance and gait improvements. The most clear-cut approach is therefore to selectively enhance cognitive capacity using seated computerized cognitive training.

### Computerized cognitive training

**Figure 4:**
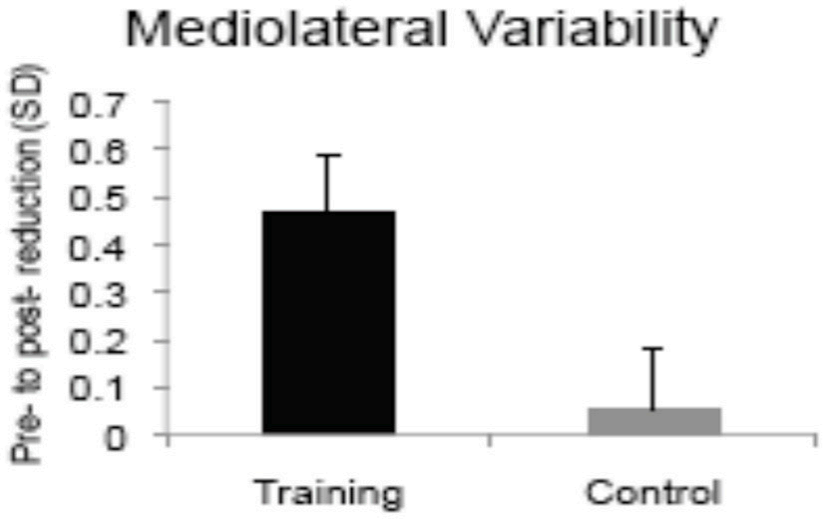
Bars show magnitude of training-related improvements in postural sway during single support balancing after computerized dual-task training vs. no-treatment [adapted from (114)]. Copyright permission not required.

An early computerized training study examined the effects of dual-task training on single and dual-task postural control (114). Healthy older adults completed five sessions of seated dual-task training involving two visual-manual reaction time tasks (40). The trained group showed pre-to-post improvements in static and dynamic balance measures given singly and with a concurrent *n*-back cognitive load, whereas the no-treatment control group showed negligible change (see Figure 4). The degree of learning in the trained cognitive task, particularly in dual-task trials, was correlated with the magnitude of improvement in postural stability, suggesting that the “active ingredient” of the training was dual-task coordination and not general processing speed. As mentioned earlier, the same DT training protocol led to increased left VLPFC activity in older adults, and reduced DLPFC activity while performing the trained task after training compared to before training (52). Future research comparing brain activity at pre- and post-training during motor and CMDT performance is needed to directly evaluate the neural overlap hypothesis, however the extant evidence of neuroplasticity is aligned with the upregulation observed in fronto-lateral brain regions during dynamic balance (105).

**Figure 5:**
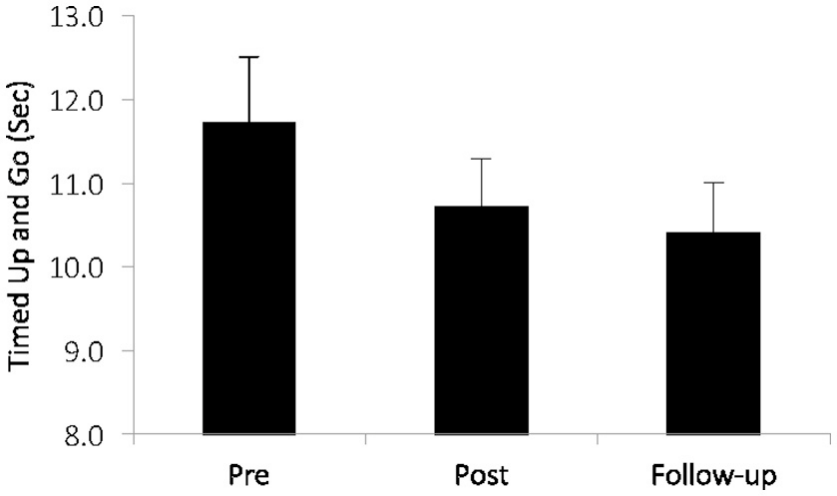
In patients with mild to moderate Parkinson's disease after executive function training [adapted from (117)]. Training-related changes over time (*p* = 035) in a clinical index of mobility: the Timed-Up-and-Go (TUG). Copyright permission not required.

Others have examined commercially available cognitive training programs targeting multiple cognitive and EF processes such as attention, visual working memory, and speed of processing, and observed greater improvements in Timed-Up-and-Go (TUG), gait velocity, and dual-task gait, compared with untrained controls (115, 116). In older fallers, cognitive training was more beneficial for TUG performance than in non-fallers (116). In low to moderate-severity patients with PD, significant improvements were found on TUG and global cognition after 36 sessions of EF training [(117), see Figure 5].

### Pharmacological and non-pharmacological stimulation

Another approach to enhancing cognitive function in order to improve mobility and CMDT is to leverage the cognitive enhancing effects of certain pharmacologic agents. For example, methylphenidate and rivastigmine apparently improve cognition and mobility and, at least among people with PD, a reduction in fall risk may be related to the effects of the drug on cognition and CMDT (118–121). While the mechanisms are likely different from that of cognitive remediation training, these studies further highlight the idea that “cognitive” interventions can enhance mobility. Similarly, recent work using non-invasive brain stimulation that targets cognitive areas also supports this idea. Indeed, a number of studies have reported that stimulation of the DLPFC either using repetitive trans-cranial magnetic (rTMS) or transcranial direct current stimulation (tDCS) enhances cogntion and reduces dual-tasks costs of balance and gait in older adults and patient groups [e.g., (122–125)].

### Exercise training

Beyond the non-motor cognitive training approach, more recent studies have added aerobic exercise training, again to enhance cognitive capacity in frontal regions and thereby free up more capacity to support motor control. Synergistic effects of combined cognitive and aerobic training were examined in a study of healthy older adults, randomized to one of four training conditions: aerobic plus EF training, aerobic plus computer lessons (cognitive placebo), EF training plus stretching (physical placebo), or cognitive placebo plus physical placebo (68, 126). Each group completed two exercise sessions and a separate computer session per week for 12 weeks. Across an array of cognitive and physical outcome measures, including CMDT gait and balance, the first three training formats yielded equivalent benefits, suggesting the absence of synergistic benefits with combined training. Similarly, cognitive training showed equivalent benefits to aerobic exercise on spontaneous walking speed (127). Using similar training tasks (aerobic cycling, computerized DT training), subsequent research with healthy older adults has examined whether delivery format (sequential versus simultaneous) yields differential benefits to CMDT. The sequentially trained group showed greater gains in working memory outcomes than the simultaneously trained group (128), whereas both groups showed similar CMDT Sit-to-Stand improvements [(129); see also (130)].

**Figure 6:**
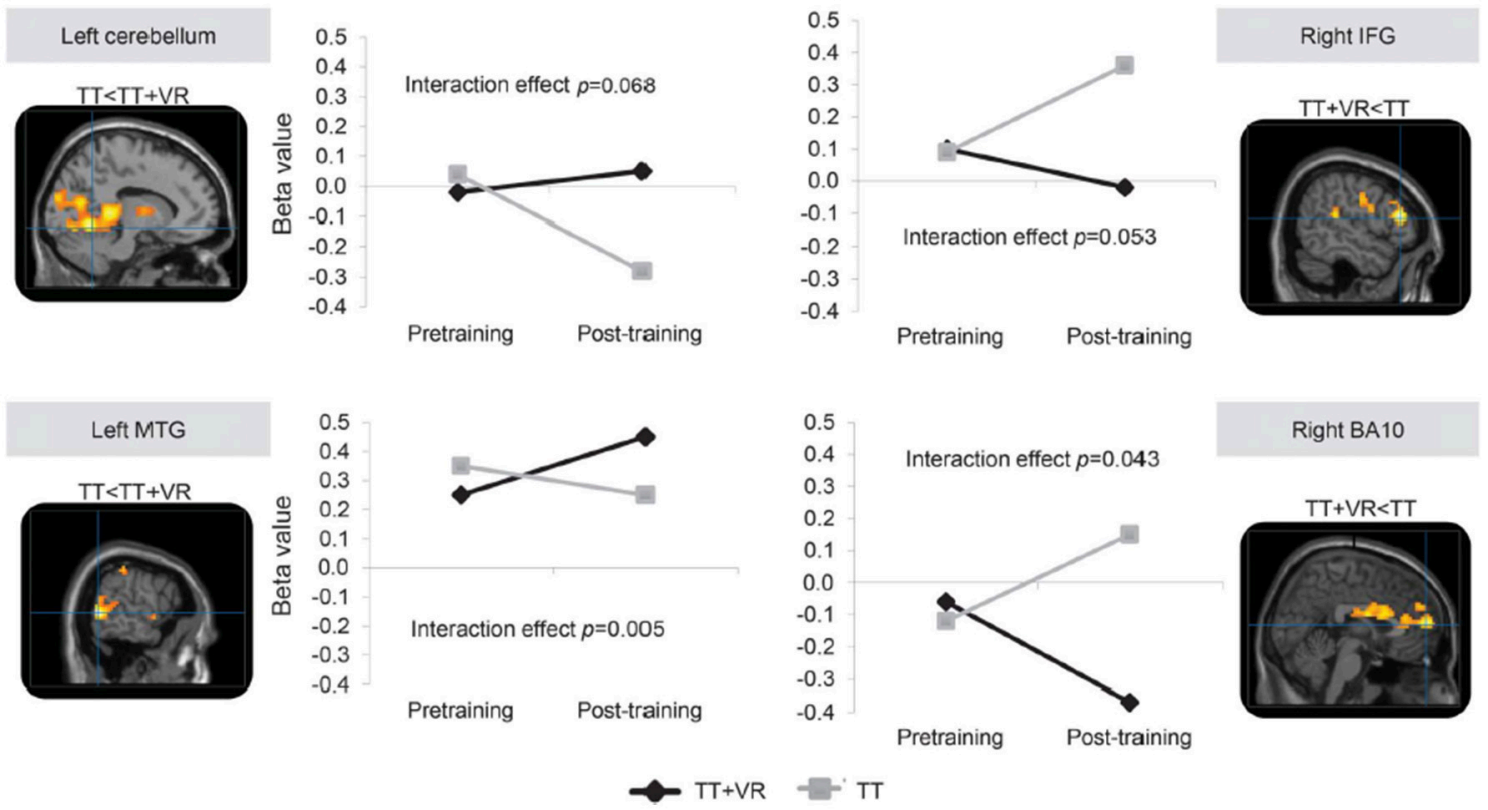
fMRI data adapted from Maidan et al. (132). Training-specific differences in brain activation during obstacle negotiation after two interventions. The images present the 4 brain areas with different patterns of activation after training between the 2 training arms, while the corresponding graphs show the changes in mean β values for voxels in each of these 4 brain areas before and after training. *P*-values are from mixed model analyzes and represent the interaction between time (pre- vs. post-training) and training arm (treadmill training, TT vs. treadmill training with virtual reality, TT + VR). BA, Brodmann area; IFG, inferior frontal gyrus; MTG, middle temporal gyrus. Figure reproduced by permission of Wolters Kluwer Health Inc. (4386371208392).

A final category of multi-modal training that contains a cognitive component is exergaming or virtual reality treadmill training. In contrast to the foregoing DT training approaches, this category reflects training activities in which the cognitive processing is integral to the motor task. For example, a randomized controlled study of older adults with a history of falls based on largely motor (i.e., PD patients), cognitive (i.e., people with mild cognitive impairment) and a mixed background (i.e., idiopathic fallers), compared the impact of treadmill training to the impact of treadmill training augmented with non-immersive virtual reality (VR) for 6 weeks (3 times per week), that targeted both cognitive aspects of safe ambulation (e.g., EF) and mobility (131). Falls, a problem presumably related to motor and cognitive function and to CMDT, were significantly reduced in the group who trained with treadmill training that included the targeting of cognitive aspects. More specifically, 6 months after the end of training, the incident rate of falls was significantly lower in the treadmill training plus VR group than in the treadmill training group (incident rate ratio 0.58, 95% CI 0.36–0.96; *p* = 0.033). Moreover, although usual single task walking improved similarly in both groups, walking under challenging conditions (e.g., obstacle negotiation variability and clearance) improved more in those who also received the cognitive training. Interestingly, in a subset of subjects with PD who underwent imaging before and after the training, fMRI and fNIRS results supported the added value of the cognitive component (see Figure 6). For example, among the subjects who underwent treadmill training alone, prefrontal activation during dual-task walking and obstacle negotiation increased after training, while in the combined training arm, activation decreased (132, 133). These findings support the idea that cognitive-motor training can reduce the need for cognitive compensation and the impact of CMDT, improve performance (more than motor training alone), and lead to changes in brain function and activation patterns.

## Conclusions and recommendations

In the foregoing review, we have discussed aspects of the cognitive neuroscience of aging that are pertinent to the study of gait and balance in aging. Accordingly, we highlight several key points that might inform future studies of CMDT. First, the research on brain aging suggests a more accelerated age-related decline of gray and white matter in anterior structures than posterior structures, with white matter changes preceding gray matter atrophy, both leading to alterations within and across brain networks. Models of cognitive and brain aging suggest that compensatory prefrontal activity, or neural recruitment, may occur in the face of structural and functional declines in response to task demands that exceed available resources. However, the same prefrontal regions are also implicated in supporting age-related declines in sensory, cognitive, and motor domains. Thus, the aging of multiple systems implies competition for common neural structures and potential tradeoffs when older adults multi-task or when any one domain of functioning becomes more challenging (e.g., with sensory or cognitive impairment, with reduced mobility). We add that the same patterns of interference and tradeoff may occur in young adults, given sufficiently challenging task demands.

The principle of neural overlap applies both to the issue of dual-task interference, and to the issue of training-related transfer. In the case of CMDT performance, we argue that consideration of the neural underpinnings of single-task cognitive and motor conditions, compared to the CMDT condition, can elucidate patterns of dual-task costs and facilitation. Similarly, consideration of the neural underpinnings of cognitive training or combined multimodal training should provide more direct evidence for the type of cognitive scaffolding and scaffolding enhancement that, to date, has been examined primarily in the realm of cognitive outcomes. Again, following the principle of neural overlap, it would be expected that training protocols that target discrete cognitive processes are likely to benefit related cognitive performance, either tested singly or in the context of CMDT performance. In contrast, the same cognitive training protocols might also indirectly benefit single- and dual-task motor performance by enhancing the capacity for compensatory cognitive or neural recruitment. Future studies should include neural outcome measures at pre- and post-training if feasible, in addition to independent behavioral indices of the targeted cognitive processes to be trained. Associating the magnitude of cognitive and neural plasticity to the magnitude of improvement in motor and CMDT performances would provide a more detailed understanding of the “active ingredients” underlying cognitive training effects.

Returning to the question posed in the introductory section, the extant research on neurocognitive aging and plasticity suggests that we cannot assume that the nature of cognitive involvement in postural control and gait is the same in younger and older adults. The available studies that combine age-comparisons, behavioral and functional neuroimaging measures of single-task cognitive, single-task motor, and dual-task performance, are few, and even more scant if one includes cognitive training and pre- and post-training imaging data.

## Author contributions

KL and JH wrote the original draft of the manuscript. AM, IM, and LB contributed additional material. All co-authors contributed to the conceptualization of the manuscript.

### Conflict of interest statement

The authors declare that the research was conducted in the absence of any commercial or financial relationships that could be construed as a potential conflict of interest.

## Abbreviations

ACC: Anterior cingulate cortex
BDNF: Brain derived neurotrophic factor
CMDT: Cognitive-motor dual-tasking
DLPFC: Dorsolateral prefrontal cortex
DT: Dual task
DTC: Dual-task cost
DTI: Diffusion tensor imaging
EEG: Electroencephalography
EF: Executive function
FA: Fractional anisotropy
HAROLD: Hemispheric asymmetry reduction in older adults
HbO_2_: Oxy-hemoglobin
HbR: Deoxy-hemoglobin
IGF: Insulin-like growth factor
MRI: Magnetic resonance imaging
NIRS: Near-infrared spectroscopy
PD: Parkinson's disease
PET: Positron emission tomography
PFC: Prefrontal cortex
SMA: Supplementary motor area
STAC: Scaffolding theory of cognitive aging
TUG: Timed-up-and-go
VEGF: Vascular endothelial growth factor
VLPFC: Ventrolateral prefrontal cortex
VR: Virtual reality
WMH: White matter hyperintensities.
